# A Ubiquitin-Based Module Directing Protein–Protein Interactions in Chloroplasts

**DOI:** 10.3390/ijms242316673

**Published:** 2023-11-23

**Authors:** Yinjie Guo, Qiuxin Li, Daili Ji, Lijin Tian, Jörg Meurer, Wei Chi

**Affiliations:** 1 Photosynthesis Research Center, Key Laboratory of Photobiology, Institute of Botany, Chinese Academy of Sciences, Beijing 100093, China; guoyinjie@ibcas.ac.cn (Y.G.); liqiuxin@ibcas.ac.cn (Q.L.); jidaili@ibcas.ac.cn (D.J.); ltian@ibcas.ac.cn (L.T.); 2 University of Chinese Academy of Sciences, Beijing 100049, China; 3 Faculty of Biology, Plant Molecular Biology, Ludwig-Maximilians University, D-82152 Munich, Germany; meurer@bio.lmu.de; 4 The Innovative Academy of Seed Design, Chinese Academy of Sciences, Beijing 100101, China

**Keywords:** protein–protein interactions, ubiquitin-based module, BiFC, chloroplast, thylakoid proteins

## Abstract

A promising approach for the genetic engineering of multiprotein complexes in living cells involves designing and reconstructing the interaction between two proteins that lack native affinity. Thylakoid-embedded multiprotein complexes execute the light reaction of plant photosynthesis, but their engineering remains challenging, likely due to difficulties in accurately targeting heterologous membrane-bound proteins to various sub-compartments of thylakoids. In this study, we developed a ubiquitin-based module (Nub–Cub) capable of directing interactions in vivo between two chloroplast proteins lacking native affinities. We applied this module to genetically modify thylakoid multiprotein complexes. We demonstrated the functionality of the Nub–Cub module in the model organism *Arabidopsis thaliana*. Employing this system, we successfully modified the Photosystem II (PSII) complex by ectopically attaching an extrinsic subunit of PSII, PsbTn1, to CP26—a component of the antenna system of PSII. Surprisingly, this mandatory interaction between CP26 and PsbTn1 in plants impairs the efficiency of electron transport in PSII and unexpectedly results in noticeable defects in leaf development. Our study not only offers a general strategy to modify multiprotein complexes embedded in thylakoid membranes but it also sheds light on the possible interplay between two proteins without native interaction.

## 1. Introduction

In both eukaryotic and prokaryotic cells, the majority of proteins do not act in isolation, but instead engage in interactions with one or more other proteins to create more-extensive protein complexes. Protein–protein interaction has consistently remained a fundamental focus in biological science, leading to the development of numerous methods for its investigation [1]. The utilization of protein modules has enabled the design and reconstruction of protein–protein interactions lacking native affinities, a technique widely employed in synthetic biology [2,3]. Although native chemical ligation is an elegant method for linking proteins, it appears to have limitations in terms of specificity and yield when used in living cells [4]. Recently, the SpyCatcher-SpyTag protein conjugation system has emerged as a means to generate stabilized multiprotein complexes in both in vitro and intracellular environments [5,6]. This system relies on the establishment of a stable covalent isopeptide bond between protein partners [7]. However, only a limited number of protein interactions mediated by such chemical reactions have been observed in eukaryotic cells. Additionally, there have been relatively few reports regarding universal modules for engineering protein complexes in plant cells.

Synthetic biology offers a crucial opportunity in higher plants to reengineer the light reactions of photosynthesis, which could lead to a significant improvement in the efficiency of these reactions. Such an improvement has the potential to increase biomass and crop yields [8,9]. Moreover, integrating this process with previously unconnected pathways will enable us to harness the reducing power of the light reactions to directly generate substantial quantities of valuable compounds [10,11]. To enhance the efficiency of the light reaction in photosynthesis, modification of the multiprotein complexes embedded in the thylakoid membrane is considered one of the most effective strategies. Since the light reaction relies on these complexes, altering them could lead to significant improvements in the process [8,12]. Numerous attempts have been made to introduce heterologous photosynthetic proteins into species that do not naturally possess their corresponding homolog, with the aim of improving photosynthesis. However, it appears that most instances of enhancing photosynthesis by simply expressing heterologous photosynthetic proteins have mainly been effective for soluble proteins [13,14,15,16,17,18]. Nevertheless, there have been fewer reported cases of this method being successful with membrane-bound proteins. This observation could prompt significant inquiries about the contrasting functions of chloroplast soluble and membrane-bound proteins. For example, in comparison to soluble stromal proteins, heterologous membrane-bound proteins may pose challenges in terms of being directed to specific sub-compartments of thylakoids, such as grana stacks, stroma lamellae, lumen, or stroma-exposed regions, where they would interact with their putative partners. Therefore, when designing modules that facilitate protein–protein interactions in chloroplasts, it is crucial to consider the specifics of targeting and sorting heterologous membrane-bound proteins.

Ubiquitin is a conserved protein of 76 amino acids that is usually attached to the N-terminal of proteins as a signal for their degradation. It was found that an N-terminal part (Nub, amino acids 1–34) has a high affinity for a C-terminal part of ubiquitin (Cub, amino acids 35–76), and they can spontaneously assemble into a split-ubiquitin heterodimer when expressed in the same yeast cell [19]. Building on this idea, our study developed a ubiquitin-based module that can drive in vivo interactions between two chloroplast proteins that normally lack native affinities. This system is not only useful for reconstructing chloroplast membrane-bound protein complexes but also for those of chloroplast soluble proteins. By utilizing this system, we were able to effectively alter the Photosystem II (PSII) complex in *Arabidopsis* by ectopically attaching one of the extrinsic subunits of the PSII core, PsbTn1, to CP26, a component of the PSII antenna system. Surprisingly, this mandatory interaction of CP26 and PsbTn1 had a noticeable impact on the photosynthetic electron transport rate (ETR) of PSII. In addition, this manipulation resulted in unexpected defects in the leaf development of *Arabidopsis*. Our study offers not only a general strategy to modify multiprotein complexes embedded in membranes, such as thylakoid multiprotein complexes and mitochondrial respiratory chain complexes but also a route to explore possible interactions between two proteins that lack inherent affinity. This module has the potential to be a versatile tool for studying and manipulating protein–protein interactions in the chloroplasts of plant cells. It will offer new insights into various aspects of chloroplast biogenesis and opens up avenues for enhancing photosynthesis and crop yield through synthetic biology approaches.

## 2. Results

### 2.1. The Design of a Ubiquitin-Based Module

**Figure 1:**
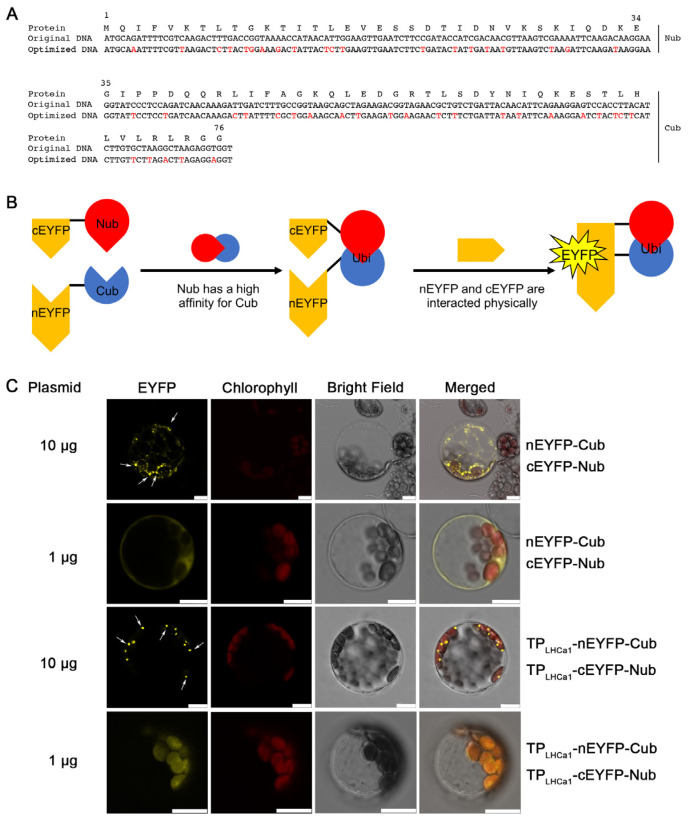
Design of Nub–Cub modules. (**A**) The original DNA sequence of *Saccharomyces cerevisiae* encoding Nub and Cub (upper lanes), and its optimized version for expression in *Arabidopsis* (lower lanes). The nucleotides with red colors indicate the changed ones in the optimized DNA sequence. The amino acids of split-ubiquitin are shown on the top. Nub and Cub represent the N-terminal (amino acids 1–34) and C-terminal (amino acids 35–76) of ubiquitin, respectively. (**B**) Schematic diagram of protein interaction mediated by Nub and Cub. Nub has a high affinity for Cub, while the N- and C-terminal parts of EYFP (nEYFP and cYFP) physically interact, thereby producing YFP signals. Nub, N-terminal of ubiquitin; Cub, C-terminal of ubiquitin; Ubi, ubiquitin. (**C**) BiFC analyses for the interactions between Cub and Nub with and without TP_LHCa1_ in *Arabidopsis* mesophyll protoplasts. TP_LHCa1_, the transit peptide of LHCa1 (amino acids 1–67 of AT3G54890). Yellow, EYFP fluorescence signals; red, chloroplast autofluorescence signals; grey, bright field. Scale bars = 10 μm. Either 10 or 1 µg of plasmids were used for transformation as indicated. The arrows indicate the distinct signals with higher amounts of plasmids.

As mentioned above, Nub and Cub fragments of yeast ubiquitin can assemble spontaneously to form a split-ubiquitin heterodimer in vivo. Our idea was to fuse Nub and Cub to two proteins that do not naturally interact. We expected that the resulting fused proteins would physically interact due to the strong affinity between Nub and Cub (Figure 1A). Due to the codon usage bias, the expression of truncated yeast ubiquitin might potentially impact gene expression levels in heterologous expression systems such as plant cells. Taking this into account, we employed codon optimization using the online software GeneTuner to optimize the codon usage of the yeast ubiquitin gene expressed in *Arabidopsis* (Figure 1B).

In a preliminary experiment, we fused Nub and Cub modules of yeast ubiquitin to the C- and N-terminal of the split YFP proteins nEYFP and cEYFP, respectively, and co-expressed these constructs in mesophyll cell protoplasts obtained from *Arabidopsis* leaves. Our aim was to obtain YFP signals arising from the interaction between nEYFP and cEYFP directed by Nub and Cub modules (Figure 1A, lane 1 and 2). Utilizing these modified and fused modules of the ubiquitin gene, we observed robust and distinct YFP signals within the cytoplasm of protoplasts, suggesting that Nub and Cub directed the interaction between nEYFP and cEYFP in plant cells (Figure 1C). This result indicated that the truncated ubiquitin modules of yeast are properly expressed in the *Arabidopsis* cytoplasm. The YFP signals obtained differed based on the amount of plasmid used for transformation; distinct signals were observed with 10 µg, while diffuse signals were seen with 1 µg (Figure 1C). With these results in mind, we proceeded to fuse the transit peptide of the LHCa1 protein (TP_LHCa1_) to the N-terminal of the split YFP proteins fused with Cub and Nub. Our expectation was that the TP sequence would guide the fused proteins into chloroplasts. We again observed prominent YFP signals that merged well with chlorophyll autofluorescence (Figure 1C, lane 3), confirming the successful functioning of the system in the chloroplasts of plant cells. However, as before, the majority of YFP signals displayed a punctate pattern within chloroplasts when the plasmid concentrations were high, whereas a better-distributed pattern was observed when the plasmid concentrations were lower (Figure 1C, lane 4). Therefore, this punctate pattern might result from the over-accumulation of fusion proteins.

### 2.2. Nub–Cub Directs the Tight Interaction of Unrelated Thylakoid Proteins

**Figure 2:**
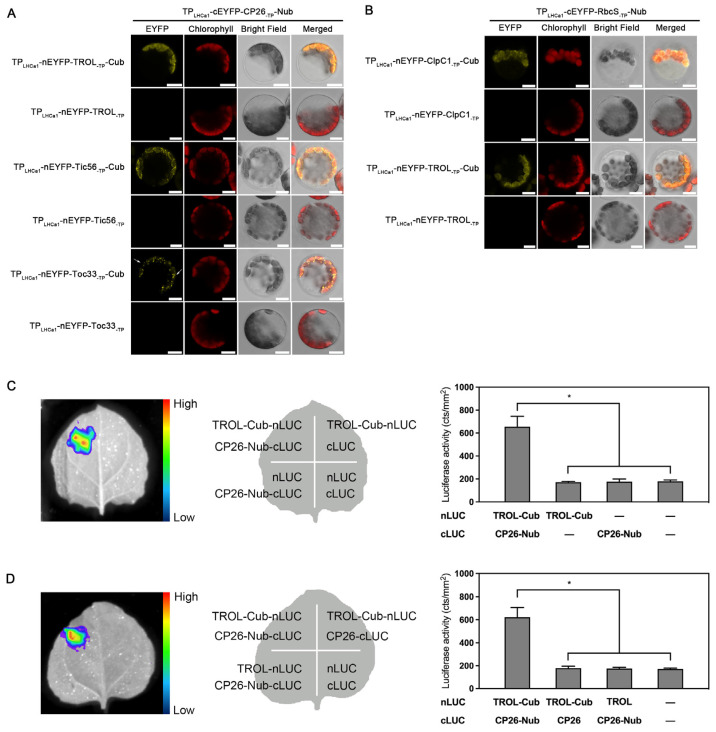
Nub–Cub directs interactions of chloroplast proteins in mesophyll protoplasts. (**A**) Nub–Cub mediated the interaction between chloroplast membrane proteins. The arrow indicate the speckles localized on the surface of chloroplasts. The co-transformation of TP_LHCa1_-cEYFP-CP26_-TP_-Nub and TP_LHCa1_-nEYFP-TROL/Tic56/Toc33_-TP_ was used for the negative control. Scale bars = 10 μm. (**B**) Nub–Cub mediated the interaction between the soluble protein and membrane proteins in chloroplasts. The experimental procedure for this step was identical to that in Figure 1, except for the type of plasmids used for the transformation process. The co-transformation of TP_LHCa1_-cEYFP-RbcS_-TP_-Nub and TP_LHCa1_-nEYFP-TROL/ClpC1_-TP_ was used for the negative control. Scale bars = 10 μm. (**C**,**D**) The luciferase complementation imaging (LCI) assay of protein–protein interaction mediated by Nub and Cub. Different plasmid combinations were co-infiltrated into *N. benthamiana* leaves and incubated for 2 days. The experimental procedure was the same in (**C**,**D)**, expect the different negative controls. Representative LUC luminescence images were shown on the left. The co-transformed plasmids in each assay are indicated in the middle diagrammatic sketch. The relative LUC activities were quantified and shown on the right. Error bars represent SD of three biological replicates. Asterisks indicate significant differences from the value of sample and control (two-sample Student’s *t*-test; * *p* < 0.05).

As an example, to investigate whether Nub/Cub can facilitate physical interaction in vivo between two unrelated thylakoid proteins containing transmembrane helices, we selected the unrelated CP26 and TROL proteins. CP26 is a minor chlorophyll-binding protein of PSII located in grana stacks, while TROL is an integral rhodanase-like membrane protein found in non-appressed stroma lamellae [20]. CP26 is known to be tightly associated with PSII super-complexes [21]. On the other hand, TROL plays a role in tethering ferredoxin: nicotinamide adenine dinucleotide phosphate (NADPH) oxidoreductase (FNR), which is located in the acceptor side of the PSI complex [22]. Due to their different localization and functions within thylakoids, CP26 and TROL are unlikely to be physically associated in vivo without assistance. We removed the sequence of the native TPs of CP26 and TROL and inserted the coding regions in-frame into the TP_LHCa1_-cEYFP-Nub and TP_LHCa1_-nEYFP-Cub constructs described in Figure 1, respectively (Figure 2). The observation of a strong YFP signal in chloroplasts co-expressing TP_LHCa1_-cEYFP-CP26_-TP_-Nub and TP_LHCa1_-nEYFP-TROL_-TP_-Cub indicates that Nub–Cub are capable of directing the interaction between CP26 and TROL (Figure 2A). Indeed, when the Cub module was removed from TP_LHCa1_-nEYFP-TROL_-TP_-Cub (the second row of Figure 2A), this interaction disappeared, suggesting that the interaction of TP_LHCa1_-cEYFP-CP26_-TP_-Nub and TP_LHCa1_-nEYFP-TROL_-TP_-Cub depends on the presence of Nub and Cub.

**Figure 3:**
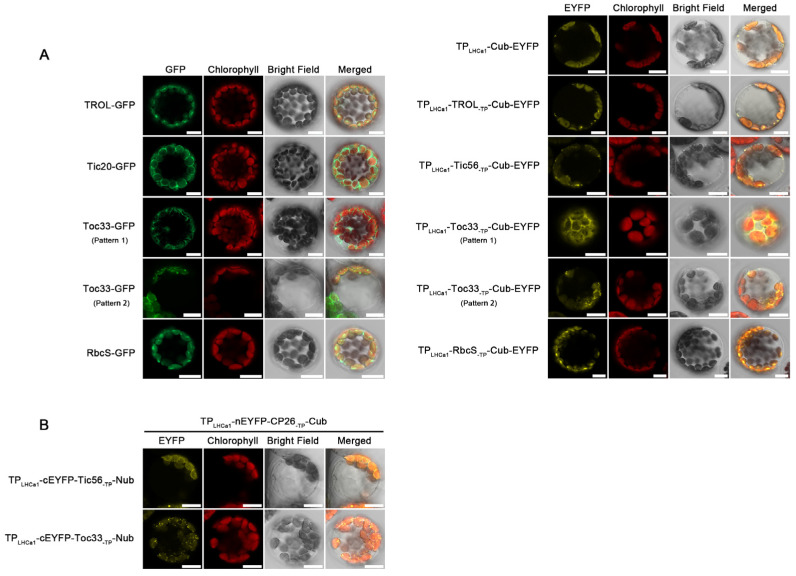
Influences of Cub module on the sub-chloroplast localization of protein–protein interactions. (**A**) Comparison of the localizations of Cub-fused proteins to those of native proteins. Left: Fluorescence signals of native chloroplast proteins fused to GFP. Two different signal patterns of Toc33-GFP proteins were shown, respectively. Right: Fluorescence signals of Cub-fused chloroplast proteins whose native TPs were replaced by TP_LHCa1_. Green, GFP fluorescence signals; red, chloroplast autofluorescence signals; grey, bright field. Scale bars = 10 μm. (**B**) The protein–protein interactions between Tic56/Toc33-Nub and CP26-Cub. The experimental procedure is same as that in Figure 2A, except the Nub and Cub module was exchanged. Scale bars = 10 μm.

Chloroplast proteins are not only associate with thylakoid membranes, but also with envelope membranes. Thus, we also investigated the Nub/Cub-mediated physical interaction of CP26 with Tic56 and Toc33 proteins located in the inner and outer envelope membrane of chloroplasts, respectively [23,24]. As expected, Nub/Cub can also direct the interaction between CP26 and the two proteins Tic56 and Toc33 in vivo (Figure 2A, lane 3 and 5). Again, both interactions disappeared when the Cub modules were removed in the TP_LHCa1_-nEYFP-Cub constructs (Figure 2A, lane 4 and 6). The YFP signal pattern observed in the Tic56 assay, which encircled the chloroplasts, resembled the pattern observed for inner and outer envelope membrane proteins in GFP assay [25,26] (Figure 3A). Since Tic56 is a well-established inner protein, this pattern likely hinted that the interaction between CP26-Nub and Tic56-Cub might occur in the inner envelope rather than thylakoid membranes. Interestingly, numerous YFP speckles appear on the “surface” of chloroplasts in the Toc33 assay, which is distinct to the pattern observed for typical outer-envelope-membrane proteins in GFP assays. Nevertheless, certain outer-envelope proteins, such as SP1 (an E3 Ubiquitin ligase), can exhibit two distinct fluorescence patterns in a single GFP assay: a circular fluorescence pattern and a speckled fluorescence pattern on the “surface” of the chloroplast [27,28]. The latter pattern closely resembles the fluorescence pattern observed in the Toc33 assay, suggesting that the interaction between CP26 and Toc33 may occur in the envelope membrane. Our findings indicate that the Nub–Cub modules are capable of effectively directing the interaction of chloroplast membrane proteins, regardless of whether they are situated in thylakoid grana stacks, stroma lamellae or in the outer or inner envelope membranes.

We next checked whether the Nub and Cub modules are also suitable for the interaction of soluble proteins within chloroplasts. Indeed, as shown in Figure 2B, the Nub–Cub modules can effectively facilitate the physical interaction of two stromal proteins, namely the protease ClpC1 [29] and the RbcS subunit of RUBISCO. In addition to the interaction between two stromal proteins, some stromal proteins directly interact with the thylakoid-embedded membrane proteins, resulting in attachment to the thylakoid membranes. Therefore, the TROL and RbcS combination was utilized as an example to explore whether Nub–Cub is capable of facilitating such protein interaction. Our findings demonstrate that the Nub–Cub system can facilitate such physical interactions between soluble and membrane proteins within chloroplasts (Figure 2B).

Given that the evidence supporting the reliability of the Nub–Cub system, as described above, is all derived from the protoplast transformation, we also employed another independent method known as luciferase complementation imaging (LCI) assay, to investigate the protein–protein interaction facilitated by Nub and Cub in tobacco leaves. By using CP26 and TROL as our experimental subjects, the LCI assay effectively demonstrated the viability of protein–protein interaction facilitated by Nub and Cub (Figure 2C).

### 2.3. Impacts of Cub Module on Sub-Chloroplast Localization

In Figure 2, when Cub was fused to TROL, Tic56, and Toc33, respectively, the protein–protein interaction occurred in the thylakoids, inner envelope, and outer envelope, respectively. Therefore, one could argue that the location of the protein–protein interaction within chloroplasts is determined by the Cub module or the Cub-fused protein. To clarify this, we initially designed a series of control plasmids (TP_LHCa1_-TROL/Tic56/Toc33_-TP_-Cub-EYFP). We then compared the localization of these fusion proteins to that of corresponding native proteins (Figure 3A). Observations revealed that the TP_LHCa1_-TROL/Tic56/Toc33_-TP_-Cub-EYFP proteins accurately targeted the thylakoid, inner envelope, and outer envelope, respectively. These sub-chloroplast locations mimic those of the native proteins (Figure 3A), indicating that Cub does not affect the sorting and targeting of fusion proteins within chloroplasts after their import from the cytoplasm to the chloroplast driven by TP_LHCa1_. Thus, Cub alone does not influence the location of the protein–protein interaction within chloroplasts. Interestingly, similar to SP1, both native Toc33 protein and modified Toc33 protein driven by TP_LHCa1_ exhibited two fluorescence patterns (circular pattern and speckled pattern). However, the underlying mechanism remains unknown.

Next, we exchanged the Nub and Cub modules in Figure 2 and examined their influence on the pattern of protein–protein interactions. As shown in Figure 3B, Tic56 and Toc33 were fused to the Nub module, while CP26 was fused to the Cub module. Interestingly, the signal patterns remained similar to those observed in Figure 2A. For instance, the speckled fluorescence pattern was also observed in the interaction between Toc33-Nub and CP26-Cub, which is identical to the pattern observed for the interaction between Toc33-Cub and CP26-Nub. Hence, the sub-chloroplast localization where protein–protein interactions occur is not determined by the Cub or Cub-fused protein.

### 2.4. The Nub–Cub System Guides Chloroplast Protein Interaction in Plants

Our next objective was to investigate whether the Nub–Cub module can direct protein interactions not only transiently but also in plants. In this assay, we selected CP26 along with PsbTn1, a 5 kDa subunit of PSII [30,31]. According to the solved structure of eukaryotic PSII complexes, PsbTn1 is extrinsically exposed to the luminal side of the eukaryotic PSII core complex [31] and intercalates between CP47 and the C-terminal region of PsbE [32]. Thus, it is unlikely that PsbTn1 directly physically interacts with CP26.

**Figure 4:**
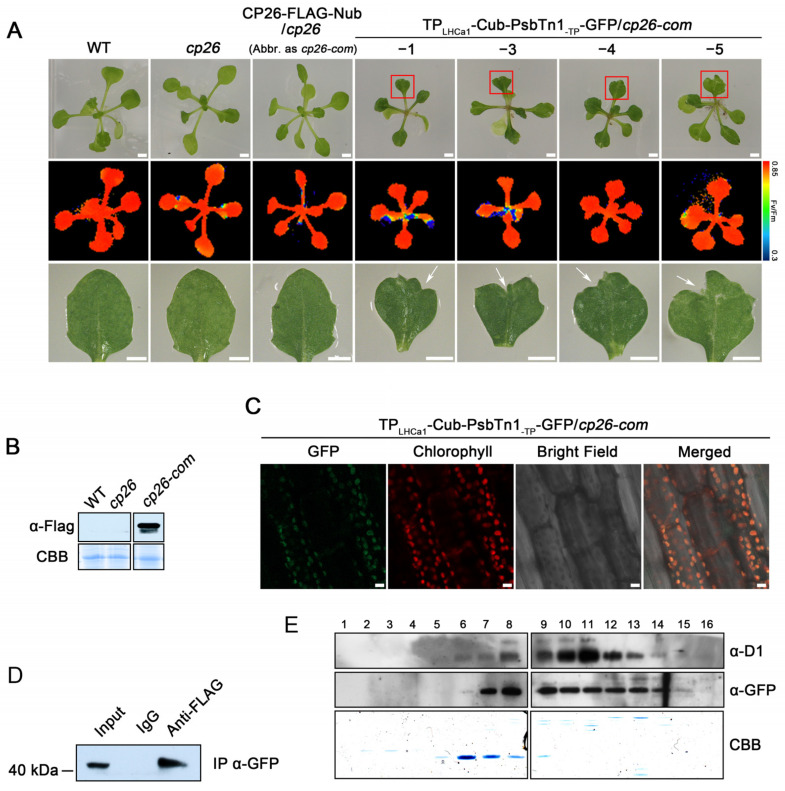
Nub–Cub directs interactions of chloroplast proteins in plants. (**A**) Phenotype of four *Arabidopsis* lines expressing CP26-FLAG-Nub and TP_LHCa1_-Cub-PsbTn1_-TP_-GFP in the cp26 background. *Arabidopsis thaliana* seedlings were grown on MS medium for 2 weeks. False-color images representing Fv/Fm ratios and the pseudocolor index are shown below the panel. The leaves with irregular margins are enlarged in the bottom panel. Scale bars = 1 mm. The red boxes and white arrows indicate the irregular leaf margins. (**B**) Immunoblotting assay of CP26-FLAG-Nub in transgenic plants lines using FLAG antibodies. (**C**) GFP assay for the expression of TP_LHCa1_-Cub-PsbTn1_-TP_-GFP in transgenic plants using confocal laser-scanning microscopy. The GFP signal was observed in the hypocotyl of *Arabidopsis* seedlings. Green, GFP fluorescence signals; red, chloroplast autofluorescence signals; grey, bright field. Scale bars = 10 μm. (**D**) Co-immunoprecipitation (Co-IP) assay of the interaction between CP26-FLAG-Nub and TP_LHCa1_-Cub-PsbTn1_-TP_-GFP. Thylakoid membrane extracts were subjected to immunoprecipitation with FLAG antibodies, and the presence of TP_LHCa1_-Cub-PsbTn1_-TP_-GFP in the immunoprecipitated pellets was tested using immunoblotting with GFP antibodies. Immunoprecipitation with the preimmunserum (IgG) served as negative control. (**E**) The comigration assay of D1 and TP_LHCa1_-Cub-PsbTn1_-TP_-GFP. A total of 200 µg thylakoid membranes were isolated, solubilized using DM, and separated with sucrose gradient sedimentation. Following centrifugation, 16 fractions were collected from the gradients and subjected to immunoblot analysis using D1 and GFP antibodies. The Coomassie brilliant blue (C.B.B.) staining gel was shown below.

We first expressed the CP26-FLAG-Nub fusion protein in the cp26 mutant background (*cp26-com*) (Figure 4A,B) followed by the introduction of the TP_LHCa1_-Cub-PsbTn1_-TP_-GFP fusion into *cp26-com* plants (Figure 4A,C). In this way, we generated several independent lines, named TP_LHCa1_-Cub-PsbTn1_-TP_-GFP/*cp26-com*, co-expressing both proteins (Figure 4A). The co-immunoprecipitation assay, using total protein extracts of leaves as starting material, showed that the FLAG antibody successfully co-immunoprecipitated the TP_LHCa1_-Cub-PsbTn1_-TP_-GFP fusion protein (Figure 4D), indicating a physical association between CP26-FLAG-Nub and TP_LHCa1_-Cub-PsbTn1_-TP_-GFP in vivo. In addition, we conducted a comigration assay to determine if the interaction between protein CP26 and PsbTn1 occurs within PSII complexes. In this assay, thylakoid membranes were isolated, solubilized using DM, and separated using sucrose gradient sedimentation. Following centrifugation, 16 fractions were collected from the gradients and subjected to immunoblot analysis using specific antibodies (Figure 4E). If the interaction between FLAG-fused CP26 and GFP-fused PsbTn1 occurs within PSII complexes, they should co-migrate with PSII core subunits such as D1 (Figure 4E). Our results demonstrated that GFP-fused PsbTn1 indeed co-migrated with D1, thereby validating our hypothesis.

**Figure 5:**
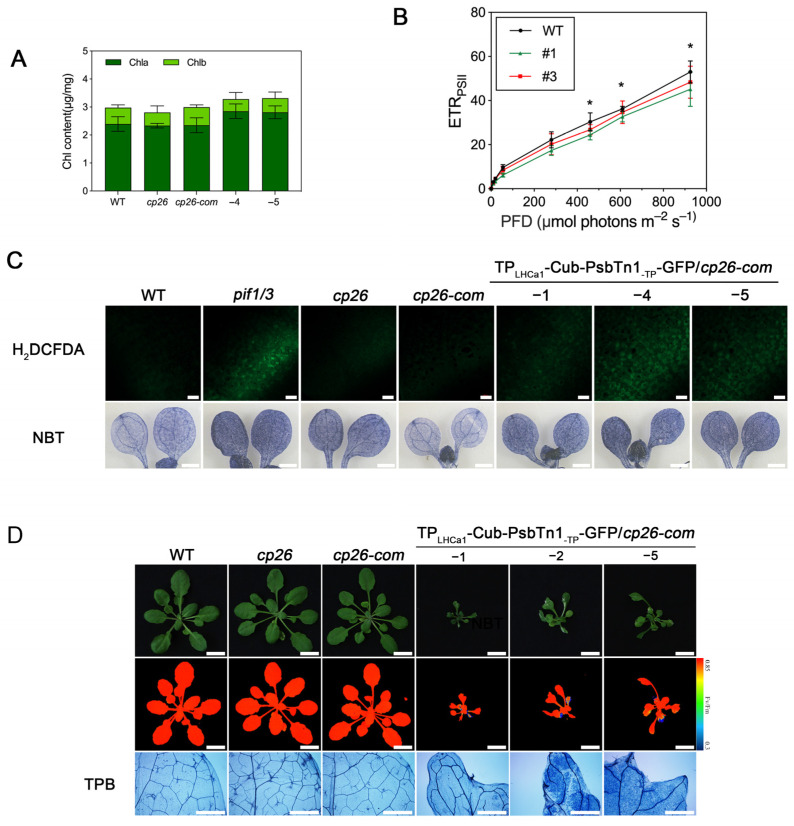
Interaction of CP26 and PsbTn1 mediated by Nub–Cub leads to ROS accumulation and cell death. (**A**) The chlorophyll contents of WT and transgenic plants co-expressing CP26-FLAG-Nub and TP_LHCa1_-Cub-PsbTn1_-TP_-GFP. Results are presented as means ± SD (*n* = 6). (**B**) Photosynthetic ETR in WT and transgenic plants. Results are presented as means ± SD (*n* = 6). Asterisks indicate significant differences from the value of sample and control (two-sample Student’s *t*-test; * *p* < 0.05). (**C**) The representative images of H_2_DCFDA and DAB staining which indicate ROS accumulation in *Arabidopsis* leaves. The *pif1/3* plant which accumulates ROS was used as a positive control. Scale bars = 100 μm (upper lane), scale bars = 1 mm (bottom lane). (**D**) Representative images of trypan blue staining (TPB) indicate cell death in *Arabidopsis* leaves. Significant mesophyll cell death (shown as blue stained cells) was observed in transgenic plants. Scale bars = 1 cm (lane 1 and 2), scale bars = 1 mm (lane 3).

Compared to WT plants, the growth of almost all leaves of transgenic lines co-expressing CP26-FLAG-Nub and TP_LHCa1_-Cub-PsbTn1_-TP_-GFP was retarded and disorderly to varying degrees (Figure 4A). The leaves of the transgenic lines with co-expressing proteins appeared to be darker green than the controls; however, we have not observed any significant changes in chlorophyll contents (Figure 5A). This growth retardation might be a result of photosynthetic dysfunctions of PSII due to the Nub–Cub mediated interaction of PsbTn1 with CP26. The Fv/Fm ratio, representing the maximum photochemical efficiency of PSII, did not appear to be affected (Figure 4A). However, we observed a clear reduction in the ETR of PSII in these plants (Figure 5B).

Intriguingly, leaves of transgenic lines unexpectedly displayed irregular leaf margins. This is in contrast to the rounded leaves of WT plants (Figure 4A). It has been proposed that ROS and its resulting programmed cell death is necessary for the progression of cellular proliferation and differentiation, thus regulating morphology during leaf development [33]. Indeed, the cell death (detected using trypan blue staining) and the increased ROS accumulation were observed in leaves with irregular margins (Figure 5C,D), suggesting the notion that ROS and cell death might contribute to the irregular leaf shape. In summary, our results showed that the formation of the CP26-PsbTn1 interaction, most likely within the PSII complex, results in obvious growth and developmental abnormalities in *Arabidopsis*. Description of the experimental results, their interpretation, as well as the experimental conclusions that can be drawn are shown below.

## 3. Discussion

While several biochemical techniques have been devised for building protein interactions and engineering multiprotein complexes, it remains unclear as to what extent these methods are effective in plants in vivo. In this study, we present a straightforward and effective approach for generating interactions between two proteins within the chloroplasts of plant cells. Because the Nub–Cub modules are relatively small, their labeling is likely to have minimal impact on the activity of the interacting proteins. The covalent isopeptide bond in the Spy-tagging system allows for protein interactions that are both irreversible and stable, even at high temperatures of up to 90 °C [34]. In contrast, the binding between Cub and Nub operates at a more physiological level.

These advantages would allow for its wide application in chloroplast biology. Considering that the presence of ubiquitin on a protein is identified by enzymes called ubiquitin-specific proteases (UBPs), which leads to the cleavage of the protein, as reviewed by Varshavsky [35], one could make an argument about the stability of this system. Despite this, there have been no reports of UBPs being present in chloroplasts, largely excluding their impact on protein stability in this context. Additionally, it remains to be tested whether this system can be applied to other organelles such as mitochondria and/or in other species.

We observed in our study that when CP26 is bound to Tic56 or Toc33, the YFP fluorescence signal is localized to the chloroplast envelope rather than the thylakoid membrane (Figure 2A). Furthermore, we ruled out the possibility that the location of CP26-Tic56 and CP26-Toc33 was related to the Cub module itself (Figure 3B). These results raise an interesting but open question: what factors determine the sub-chloroplast localization of protein–protein interactions in our system? We hypothesized that, compared to CP26, Toc33 and Tic56 might have stronger membrane-anchoring abilities, enabling them to displace CP26 from the thylakoid membranes through Nub–Cub interactions. If this hypothesis holds true, the location of the interaction between these two membrane proteins would depend on the partner with the stronger membrane-anchoring ability. Alternatively, the interactions between CP26-Tic56 and CP26-Toc33 may only occur during the import of the CP26 precursor across the chloroplast envelope; therefore, these interactions occur within the chloroplast envelope. Further studies will be conducted to clarify these questions, which will contribute to improving the broader utilization of this system.

**Figure 6:**
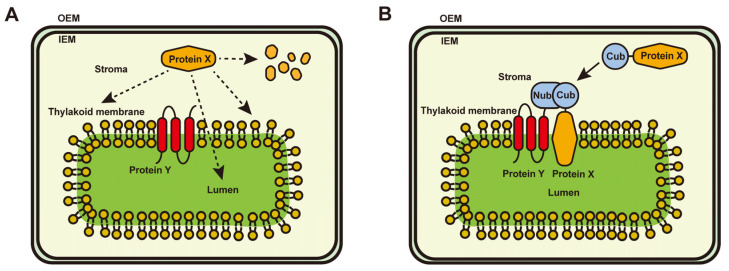
Strategy of the Nub–Cub system to target heterologous proteins to unrelated thylakoid membrane proteins. (**A**) Heterologous proteins expressed in chloroplasts (protein X, as shown in the model) might not be targeted to thylakoid membrane proteins because they are not recognized by sorting pathways. The protein X might also be degraded because it is not the targeted to the thylakoid membrane system. (**B**) An endogenous protein (protein Y, as shown in the model) and protein X were fused using the Nub and Cub tags. In this case, protein X could be targeted to the site where protein Y is localized due to the interaction between Nub and Cub.

Once the native thylakoid proteins encoded by the nucleus are imported into the chloroplasts from the cytosol, they are targeted to their correct sub-compartments through various sorting pathways [36,37]. In the case of heterologous proteins that are meant to be integrated into the thylakoid membrane, these sorting pathways may not recognize them, resulting in incorrect or no targeting of the proteins to the thylakoids, as depicted in Figure 6. It is possible that the inability of some heterologous membrane-associated proteins to function correctly within thylakoids is due to their failure to be targeted correctly by the sorting pathways. To address this issue, we have proposed a solution in which a native thylakoid protein and a heterologous protein are modified with Cub and Nub, respectively, as depicted in Figure 4. This approach is effective for both membrane-bound proteins with and without transmembrane helices. Our study presents a potential strategy for overcoming this problem. We employed the Nub–Cub module to create the interaction between CP26 and PsbTn1 in plants, resulting in reduced ETR and impaired leaf development under normal growth conditions. This outcome could mirror the consequences of an engineered CP26-PsbTn1 interaction within a living organism.

The engineered interaction of CP26 and PsbTn1 has the potential to disrupt the association between CP26 and chlorophyll or closely located proteins, leading to a decrease in the effective antenna size of PSII. This modification of the PSII antenna may contribute to the reduction in photosynthetic ETR, although there may be other explanations as well. Given that PSII significantly contributes to the overall formation of ROS in the thylakoid membrane under certain circumstances [38], the accumulation of ROS was not unexpected. We suggested that the irregular leaf shape might be due to the accumulation of ROS and cell death in those plants. Interestingly, the abnormal leaf development is limited to just one leaf per plant. According to our current knowledge, the accumulation of ROS and cell death are localized events in many mutants, rather than being uniform throughout the plant [39,40]. As a result, the resulting phenotype tends to vary among different leaves and leaf blades. For example, the *Arabidopsis acd1* mutant displays lesions caused by ROS, which mainly start at the tip of the leaf and then spread towards the leaf blade [40]. Moreover, the appearance of the lesions differs among different leaves. This observation aligns with the phenomenon we observed in this study. Nevertheless, we currently lack an understanding of how ROS precisely influences the programmed cell death and the advancement of cellular proliferation and differentiation during leaf development in those genetically modified plants. To the best of our knowledge, leaf developmental abnormalities like the ones we observed have not been reported in PSII dysfunctional mutants. Our findings suggest that the effects of the Nub and Cub modules in plants can create unique phenotypes that are rarely observed in knockout mutants or overexpression lines. This discovery presents an avenue to explore the potential interaction between two unrelated proteins.

## 4. Materials and Methods

### 4.1. Plant Material and Growth Conditions

*Arabidopsis thaliana* Columbia-0 (Col-0) WT seeds were pre-incubated in darkness at 4 °C for 48 h. Seeds were vortexed with 10% (*v*/*v*) sodium hypochlorite for 10 min, and then washed at least 5 times for surface sterilization before sowing them on a Murashige and Skoog (MS) medium containing 2% (*w*/*v*) sucrose. Seedlings were grown for 14 d in a growth chamber at 22 °C with a 12 h light/12 h dark photoperiod. When seedlings were grown in soil, we maintained the photoperiod with a photon flux density of 100 μmol m^−2^ s^−1^ at 22 °C.

### 4.2. Codon Optimization

Codon optimization of the ubiquitin gene from *Saccharomyces cerevisiae* (strain ATCC 204508/S288c) (P0CG63) was performed using an available online software GeneTuner http://180.167.32.162:8086/cool (accessed on 27 September 2020). Average GC content was adjusted from 38.2% to 29.4% and unfavorable peaks were removed. Codon usage bias was adjusted to fit the highest expression profile in the host *Arabidopsis thaliana*.

### 4.3. Plasmid Construction

The plasmid construction was based on the pSAT6 series of vectors as described [41]. Optimized DNA sequence of Nub and Cub were cloned into the EcoRI site of plasmid pSAT4A-n/cEYFP-N1 and the BamHI site of pSAT6-n/cEYFP-C1 to produce Cub-nEYFP, Nub-cEYFP, nEYFP-Cub, and cEYFP-Nub. The transit peptide of LHCa1 (AT3G54890, 1–67 amino acids) was inserted into the HindIII site of pSAT4A-Cub-n/cEYFP-N1 and pSAT4A-Nub-cEYFP-N1, the NcoI site of pSAT6-nEYFP-Cub and pSAT6-cEYFP-Nub-N1 vector, respectively.

To produce different recombinant chloroplast proteins, the coding sequence of mature TROL (AT4G01050), Tic56 (AT5G01590), Toc33 (AT1G02280), and ClpC1 (AT5G50920) proteins was PCR-amplified and inserted into the EcoRI site of pSAT6-TP_LHCa1_-nEYFP-Cub-C1 to produce TP_LHCa1_-nEYFP-TROL/Tic56/Toc33/ClpC1-Cub, respectively. The coding sequences of CP26 (AT4G10340) and RbcS (AT1G67090) were cloned into the HindIII site of pSAT6-TP_LHCa1_-cEYFP-Nub-C1 to produce TP_LHCa1_-cEYFP-CP26_-TP_/RbcS_-TP_-Nub, respectively.

### 4.4. Confocal Microscopy Imaging

All the constructs were transiently expressed in protoplasts prepared from *Arabidopsis* mesophyll protoplasts using the PEG-mediated method [42]. The plasmid concentrations for protoplast transformation range from 1 to 10 μg. The GFP/YFP fluorescence was visualized 14–18 h after transformation using a Zeiss LSM980 confocal laser scanning system (Zeiss LSM980; Carl Zeiss, Oberkochen, Germany). Chlorophyll fluorescence was excited with the argon laser at 514 nm and detected between 627 and 726 nm; YFP/GFP emission was also excited at 514 nm, and the emitted fluorescence measured between 525 and 546 nm.

### 4.5. Chlorophyll Fluorescence Measurements

Chlorophyll fluorescence of plant leaves was measured using PAM chlorophyll fluorometry [43].

### 4.6. Co-Immunoprecipitation Assay

The co-immunoprecipitation assay was performed using rosette leaves. Protein extracts (40 ng·μL^−1^, 4 mL) were divided into two equal volumes. One volume was mixed with 100 μL of pre-balanced FLAG-Nanoab-Magnetic Beads (LABLEAD Technology, Beijing, China), conjugated with protein antibodies, while the other volume was mixed with 100 μL pre-balanced Beads conjugated with preserum, following the manufacturer’s instructions. The mixtures were then incubated with gentle mixing overnight at 4 °C. After incubation, the beads were washed four times with 0.5% NP-40 (*v*/*v*), and the binding protein was released using elution buffer (137 mM NaCl, 2.7 mM KCl, 10 mM Na_2_HPO_4_, 1.8 mM KH_2_PO_4_, pH 7.4). The eluted proteins were separated on a 15% SDS–PAGE gel and subjected to immunoblotting.

### 4.7. The Luciferase Complementation Imaging (LCI) Assay

The nLUC- and cLUC-fusion constructs, along with P19, were individually introduced into *Agrobacterium* strain GV3101 and plated on an LB medium supplemented with kanamycin and rifampicin. After incubating in a shaker at 28 °C overnight, the bacteria were harvested with centrifugation at 4000 rpm for 10 min. The resulting pellets were then resuspended in a solution containing 10 mM MgCl_2_, 10 mM MES (pH 5.6), and 200 μM acetosyringone to achieve a final concentration of OD_600_ of 1.0. Volumes of *Agrobacterium* carrying the desired expression proteins A-nLUC, cLUC-Protein B, and P19 (in a ratio of 1:1:2) were mixed to create the infiltration solution. The bacterial suspensions were incubated at 28°C for 4 h and then infiltrated into the abaxial side of *N. benthamiana* leaves using a 1 mL needleless syringe. After 2–3 days of incubation, luciferase activity was measured using a NightSHADE LB 985 imaging system (Berthhold Technologies, Bad Wildbad, Germany).

### 4.8. ROS and Cell Death Detection

The H_2_DCFDA staining for ROS detection was performed as described previously [44]. The seedlings were stained for 20 min in 50 mM PBS buffer (pH 7.4), 50 mM H_2_DCFDA, and 0.004% Triton X-100 in the dark. The seedlings were washed 3 times with 50 mM PBS buffer (pH 7.4) and inspected with a confocal laser scanning microscope (Zeiss LSM980). Superoxide accumulation was detected by staining with NBT (LABLEAD Technology, Beijing, China). Stained leaves were boiled in acetic acid:glycerol:ethanol (1:1:3 [*v*/*v*/*v*]) and photographed. Trypan blue staining for cell death was performed as described [45].

### 4.9. The Comigration Assay

The comigration assay was performed according to the method described by Peng et al. [46]. Briefly, 200 µg of thylakoids extracted from intact chloroplast membranes were solubilized with 1% α-DDM for 10 min. Subsequently, they were centrifuged at 10,000 rpm for 10 min at 4 °C. The supernatant was then placed on a sucrose-density gradient and ultracentrifuged at 38,000 rpm for 18 h at 4 °C. Sixteen fractions were collected from the top to the bottom of the gradient, and the proteins in each fraction were separated with SDS-PAGE and characterized using immunoblot analysis.

### 4.10. Accession Numbers

Sequence data from this article can be found under the following accession numbers in the TAIR library for *Arabidopsis* genes: TROL (AT4G01050), Tic56 (AT5G01590), Toc33 (AT1G02280), ClpC1 (AT5G50920), CP26 (AT4G10340), and psbTn1 (AT3G21055).

## 5. Conclusions

The ubiquitin-based module developed in this study can direct interactions in vivo between two chloroplast proteins without native affinities. In addition, this system opens a new avenue to generate unique phenotypes rarely observed in traditional genetic modification approaches, such as knockout mutants or overexpression lines. While the system has demonstrated its efficacy in *Arabidopsis*, further exploration is needed to assess its potential applicability in other organelles or species. Future research should focus on a deeper understanding of the underlying mechanisms and the broader utility of this method in various biological processes.

In theory, this module has the ability to facilitate the physical interaction of any known/unknown chloroplast proteins, which can be used to modify the components of protein complexes in chloroplasts. Hence, it is anticipated that this module will be extensively utilized in manipulating various aspects of the photosynthesis process, particularly the light reaction within thylakoids, with the goal of enhancing photosynthesis and crop yield.

## Data Availability

Data are contained within the article.
